# Embryonic Surface Ectoderm-specific Mitofusin 2 Conditional Knockout Induces Congenital Cataracts in Mice

**DOI:** 10.1038/s41598-018-19849-2

**Published:** 2018-01-24

**Authors:** Jiangyue Zhao, Xinwei Wu, Danhong Wu, Yinhui Yu, Yibo Yu, Yao Wang, Qiuli Fu, Jinsong Zhang, Ke Yao

**Affiliations:** 1 The Department of ophthalmology, Eye center of the 2nd Affiliated Hospital, Medical College of Zhejiang University, Hangzhou, 310009 China; 2 The Department of ophthalmology of the 4th Affiliated Hospital, China Medical University, Shenyang, 110005 China; 3 Department of Neurology, Shanghai fifth People’s Hospital, Fudan University, Shanghai, 200240 China

## Abstract

Inherited mitochondrial mutations can result in mitochondrial dysfunction or stochastic oxidative damage. Cumulative mitochondrial damage is an important factor in age-related disorders, such as cataracts and macular degeneration. *Mfn2* mediates the fusion of mitochondria and contribute to the dynamic balance between fusion and fission that determines mitochondria morphology. We report here that conditional loss of *Mfn2* function in the head surface ectoderm leads to a range of congenital eye defects, including small, opacified lens and small eyeball in the most severe phenotypes. The *Le-Cre* transgenic mouse line and *Mfn2 flox* mouse line were used in this study to generate *Mfn2* conditional knockout mice. Our study revealed *Mfn2* gene function in lens development and addressed the relationship between the mitochondria and lens transparency. Conditional loss of *Mfn2* affected lens epithelium cell proliferation, apoptosis and ultrastructure of mitochondria. We conclude that proper development of the lens and lens transparency depend on normal *Mfn2* gene function.

## Introduction

Mitochondria are double membrane-bound organelles in eukaryotic organisms that are crucial in determining cell fate. Their functions include generating ATP and regulating apoptosis and Ca^2+^ homeostasis^1,2^. Mitochondria continually fuse and divide, and their quality, distribution, size, and motility are finely tuned. Mitochondrial fusion and fission directly influence mitochondrial function, such as metabolism, apoptotic and necrotic cell death, autophagy, muscular atrophy and cell migration^3,4^. Recent evidence indicates that mitochondrial dynamics influence complex signaling pathways, affect gene expression and define cell differentiation^5,6^. Mitochondria are critical for ocular function. Mitochondrial dysfunction can occur as the result of inherited mitochondrial mutations (e.g., Leber’s hereditary optic neuropathy) or stochastic oxidative damage, which leads to cumulative mitochondrial damage and is an important factor in age-related disorders, such as cataracts and macular degeneration^7–11^.

Mitofusins, such as mitofusin 2 (*Mfn2*), mediate the fusion of mitochondria and thereby contribute to the dynamic balance between fusion and fission of mitochondria^12^. *Mfn2*, encoded by nuclear DNA, is primarily localized in the outer mitochondrial membrane and is involved in its rearrangement. Moreover, *Mfn2* is also present in the endoplasmic reticulum (ER) membrane and plays a role in ER interactions with mitochondria. *Mfn2* deficiency has been shown to impair mitochondrial function^13–17^.

Congenital cataract is the leading cause of childhood blindness with a prevalence of 1–6 cases per 10,000 live births in industrialized countries and 5 to 15 per 10,000 in underdeveloped areas of the world. About 20,000 to 40,000 new cases of bilateral congenital cataracts are diagnosed each year worldwide. Approximately 70% of the congenital cataracts involve the lens alone^18^. Hereditary cataracts account for a large portion of congenital cataracts. Development of the human lens requires a tightly regulated sequence of events and the interplay of many genes. Mutations in these genes negatively affect the structure and transparency of the lens, leading to cataracts. To date, the mechanisms involved in the development of congenital cataracts have not been fully elucidated.

Herein, we demonstrate in mice that an *Mfn2* gene conditional knockout could lead to congenital cataracts due to mitochondrial dysfunction in lens cells. We also show that ROS induced mitochondrial malfunction when *Mfn2* was knocked out. These results suggest a novel mechanism implicating the involvement of mitochondria in lens metabolic function and the development of congenital cataracts.

## Results

### Generation of head ectoderm-specific *Mfn2* conditional knockout mice

**Figure 1:**
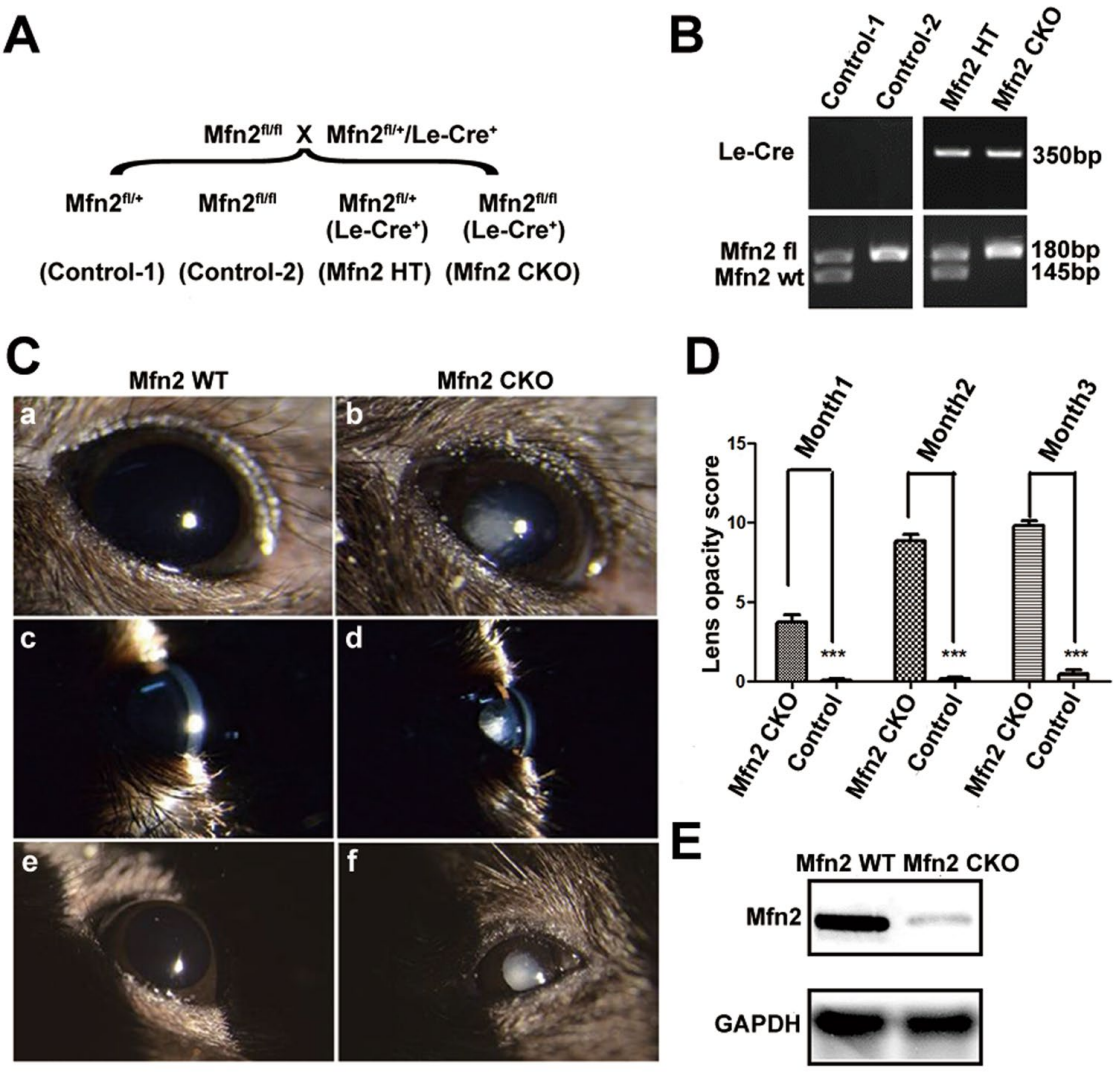
Generation of head ectoderm-specific Mfn2 conditional knockout mice. (**A**) Breeding scheme for the generation of *Mfn2 CKO* mice by crossing *Mfn2fl/fl* and *Le-Cre* mice. (**B**) Genotypes of fetal mice by tail genomic DNA PCR. Detection of Le-Cre and Mfn2 allele by genotyping PCR. The fragment of 350 bp indicates Le-Cre, the fragment of 180 bp indicates Mfn2 floxed allele and the 145 bp fragment indicates wild-type Mfn2 gene. (**C**) Phenotype of *Mfn2 CKO* and *Mfn2 WT* mice photographed by diffused light and slit light. *Mfn2 CKO* mice had congenital cataract and small eye compared with control. (**D**) Evaluation of the rate of cataract formation and opacity score in *Mfn2 CKO* and age-matched WT mice. Opacity was quantified by using the LOCS III system, and the sum of the scores were used to analyze the severity of the cataract. The same ophthalmologist gave the cataract scores without prior knowledge of the mouse genotype. ***p < 0.001 versus age-matched WT. (**E**) Western Blot for confirming the *Mfn2* protein level in *Mfn2 CKO* and *Mfn2 WT* mice.

Embryos homozygous for a germ line deletion of *Mfn2* arrest at gastrulation^19^. Therefore, we examined the offspring of *Mfn2*^*fl/fl*^ mice^20^ that also expressed a Cre transgene under the control of the Pax6 P0 promoter. In these mice, Cre is expressed in the head surface ectoderm and lens placode by Embryonic day (E) 9.5. Timed mating between *Mfn2*^*fl/fl*^ and *Mfn2*^+*/fl*^*/Le-Cre*^+^ mice generated head surface ectoderm-specific Mfn2 conditional knockout (*Mfn2 CKO*) mice (*Mfn2*^*fl/fl*^*/Le-Cre*^+^), heterozygous *Mfn2* knockout (HT) mice *(Mfn2*^*fl/*+^*/Le-Cre*^+^), and control mice (*Mfn2*^*fl/*+^ or *Mfn2*^*fl/fl*^) (Fig. 1A). Mice that were homozygous for the floxed allele would be expected to lack functional *Mfn2* in the lens and ocular surface epithelia. We confirmed the *Mfn2* protein almost diminished in the lens epithelium cell in P60 *Mfn2 CKO* mice, which proved that *Mfn2* was efficiently conditional knockout (Fig. 1E, Supplement Fig. 1). In order to demonstrate Cre-mediated recombination of the floxed *Mfn2* allele, genomic DNA isolated from the tail of a mouse fetus was subjected to PCR-based genotype analysis (Fig. 1B, Supplement Fig. 3). Since *Mfn2fl/fl* and *Mfn2fl/*+ embryos are morphologically indistinguishable from wild-type (*Mfn2*+/+) embryos, they were all classified as normal wild-type (WT) controls in this study.

### Targeted deletion of *Mfn2* lead to congenital cataracts with microphthalmia

*Mfn2 CKO* mice had postnatal congenital cataracts and microphthalmia compared to the control littermates (Fig. 1C). The eyelids and ocular surface epithelia of these mice appeared normal by gross inspection at eye opening. Enucleated eyes of *Mfn2 CKO* mice at 1 month after birth showed consistent microphthalmia comparing to the eyes of control littermates, and nearly 84% developed cataracts (Fig. 1C). Examination of eye specimens from the *Mfn2 CKO* mice from E14 to 12 weeks (N = 32) revealed that microphthalmia and cataracts were not severe during the embryo stage, but obvious after delivery and progressively worsened with age.

Slit lamp images of representative eyes from both 1- and 3-month-old CKO and control mice are shown in Fig. 1c. In general, lens opacity in the *Mfn2 CKO* groups first appeared in the nuclear or posterior subcapsular regions and spread out rapidly. As shown in Fig. 1C, lenses had slight opacity in CKO mice at 1 month old, and control littermates had transparent lenses at the same age. CKO animals exhibited a rapid loss of transparency and became severe cataractous at 3 months after birth compared to control mice that were rarely cataractous at the same age (Fig. 1C). When examining the severity of cataracts using an opacity score from 0 to 12, the two mouse populations showed significant difference, as did the formation of cataract. As shown in Fig. 1D, the CKO group showed a lens opacity score of 3.75 ± 0.53 at 1 month which steadily increased to 9.83 ± 0.94 at 3 months. Similar to the presence of cataract, the control group had slower development of lens opacity with scores of 0.08 ± 0.29 at 1 month and 0.50 ± 0.80 at 3 months. In each age group, cataract development in the control mice was far lower than in CKO animals. Opacity scores in the young mice increased predominantly due to increases in nuclear, cortex and post-subcapsular opacity.

### Normal *Mfn2* transcript in lens development

**Figure 2:**
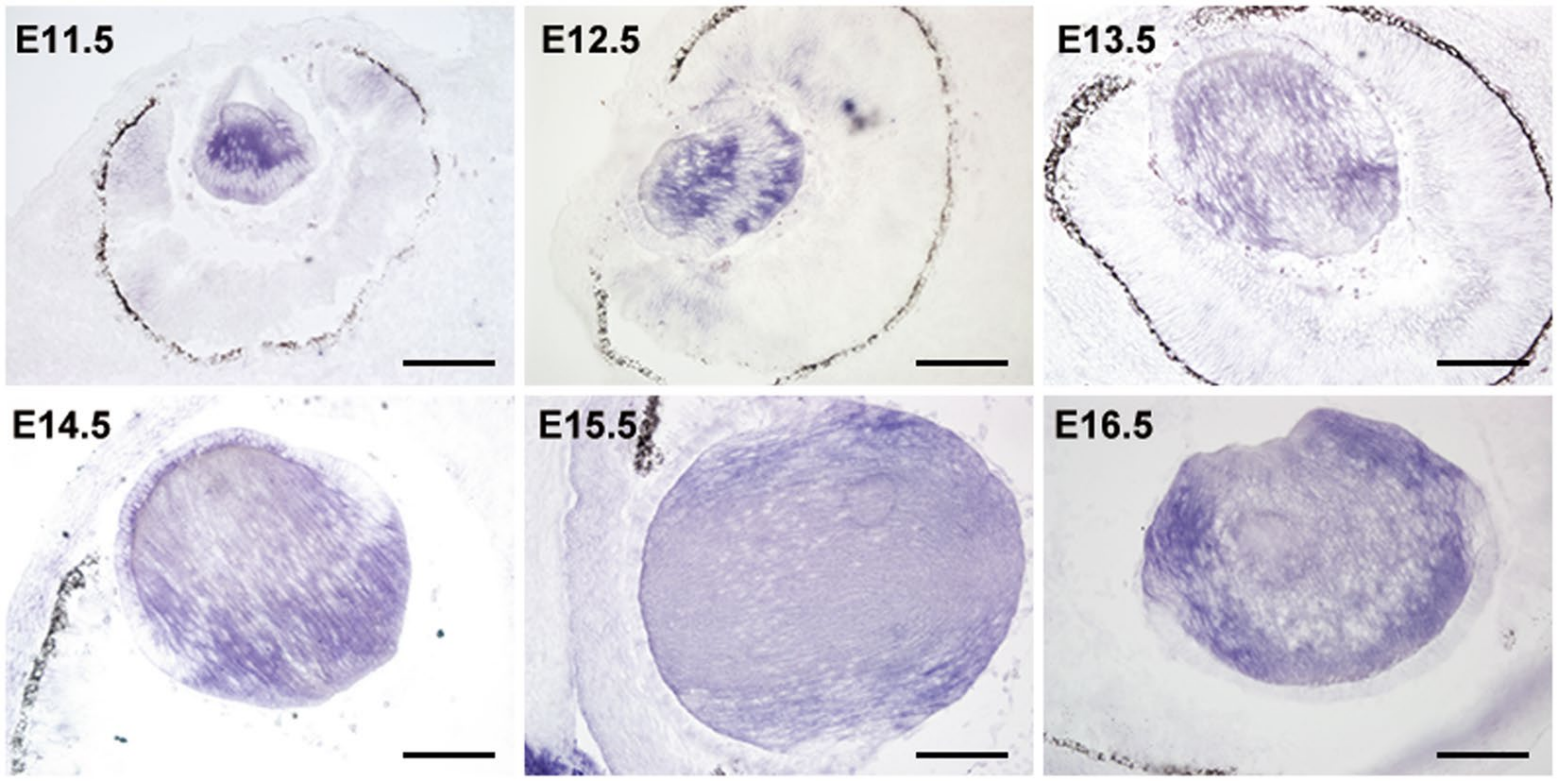
Normal *Mfn2* transcript spatiotemporal location during lens development. (**A**) *Mfn2* transcript were present in low level at lens epithelium cells and high level at the posterior portion of the lens vesicle at E11.5. (**B**–**F**) Intense *Mfn2* transcript was localized throughout lens fiber cells and the transcript was weak in the lens epithelium cell (E12.5-E116.5). Scale bars: 100um (E11.5-E15.5), 150um (E16.5).

*Mfn2* transcripts were first detected at the early lens vesicle stage (E10, Supplement Fig. 2). After that, *Mfn2* transcripts were present at low levels in lens epithelial cells and moderate to high levels in the posterior portion of the lens vesicle, which were prepared to differentiation (Fig. 2). In differentiated and elongated primary lens fiber cells (E12.5), intense *Mfn2* transcript was localized throughout the cells and expression was weak in the proliferating lens epithelium (Fig. 2). A weak signal was observed in corneal epithelium cells after E14.

### Lens development in *Mfn2 CKO* mice

**Figure 3:**
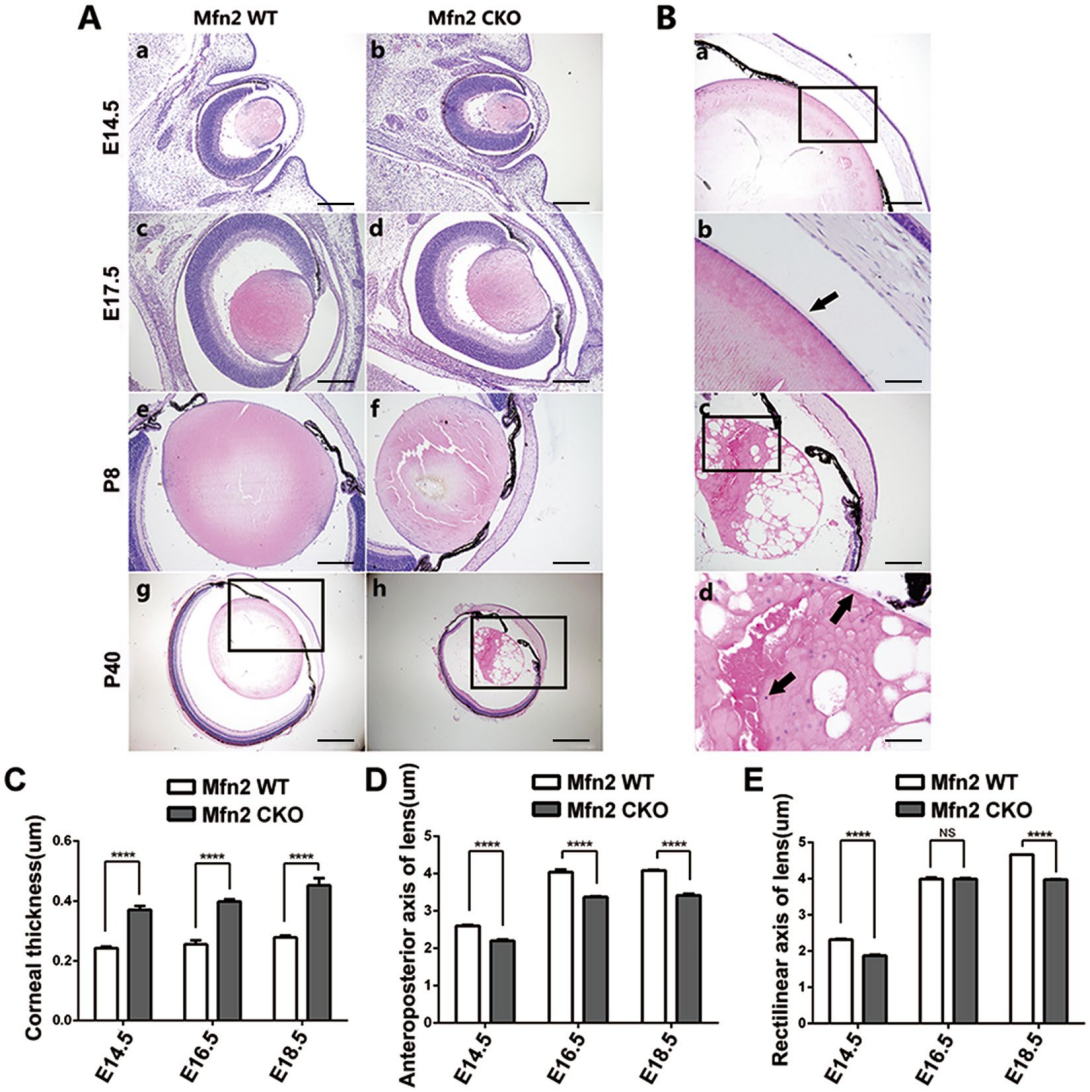
Lens development in *Mfn2 CKO* mice from E14.5 to P40. (**A**) Subtle changes in the eyes of *Mfn2* conditional knockout mice since E14.5. Lens size in the *Mfn2 CKO* mice appeared to be slightly smaller, but the complete structure was fully developed. (**B**) Histological analysis of the eyes at postnatal day 8 showed a distinct, thickened cornea, smaller lens, mild lens opacity, and central cortex vacuole in the *Mfn2 CKO* mice compared to the controls. The nuclear of lens cell distributed dispersedly around the lens equator and the back of lens cortex (arrow). At P40 lens opacity and the cortex vacuole were more severe with age (arrow). (**C**) Column diagram of the central corneal thickness of *Mfn2 CKO* and *Mfn2 WT* mice. Corneal thickness was significantly increased in *Mfn2 CKO* mice. (**D**) Column diagram of the lens anteroposterior axis length of *Mfn2 CKO* and *Mfn2 WT* mice. There were statistical significant differences of anteroposterior diameter from E14.5 to E18.5 between the two groups. (**E**) Column diagram of the lens horizontal axis length of *Mfn2 CKO* and *Mfn2 WT* mice. There were statistically significant differences of horizontal lens diameter at E14.5 and E18.5 between the two groups. (n = 5, *P < 0.05, **P < 0.001, ***P < 0.0001, ****P < 0.00001). Scale bars: (**A**) 40um (a–f), 100 um (g,h); (**B**) 20 um (a,c), 10 um (b,d).

Subtle changes in the eyes of *Mfn2* conditional knockout mice were first detected as early as E14.5 in the lens compared with the littermate controls (Fig. 3A). Examination of histological sections revealed that lens size in the *Mfn2 CKO* mice appeared to be slightly smaller, but the complete structure was fully developed (Fig. 3A). Although the lens vesicle was smaller than normal, lens fiber cells were arrayed in an orderly manner in the vesicle. Histological analysis of the eyes after postnatal day 8 day showed a distinct, thickened cornea, lens cell scattered around equator, smaller lens, slight center lens opacity, and cortex vacuole in the *Mfn2 CKO* mice compared to the controls (Fig. 3A). Corneal thickness was increased mainly in the stromal layers, but lens opacity and the cortex vacuole were more severe with age after the eyelids opened at postnatal day 14 (Fig. 3A). At postnatal day (P) 40, shrunk cataractous lens full of vacuole was observed. The lens epithelial cell below capsule organized loosely and distributed disorderly in a representative eye (Fig. 3B). Lens size was quantified by measuring the anteroposterior and horizontal diameters in both groups from E14.5 to E18.5 (Fig. 3D,E). There were statistically significant differences of horizontal lens diameter at E14.5 and E18.5 between the two groups except at E14.5 (P < 0.05). And the anteroposterior diameter length of *Mfn2 CKO* mice were statistical significant small from E14.5 to E18.5 compared with *Mfn2 WT* mice (P < 0.05). The corneal thickness from E14.5 to E18,5 were quantified and compared between the two groups. Corneal thickness was significantly increased in *Mfn2 CKO* mice (Fig. 3C).

### Abnormal cell proliferation in the lens of *Mfn2 CKO* mice

**Figure 4:**
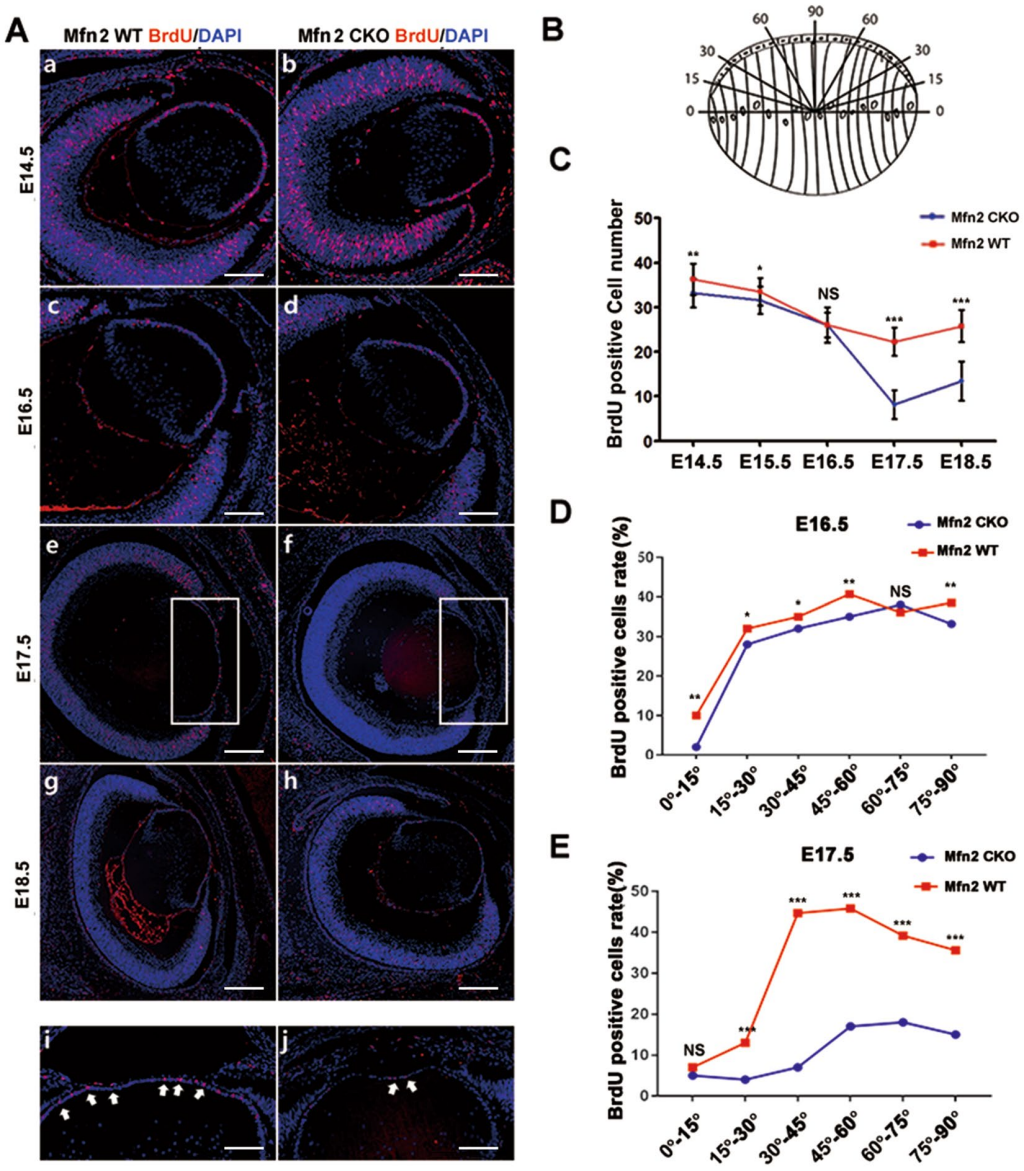
BrdU labelling in the lens of *Mfn2 CKO* mice and counting of proliferation cells at each development stage. (**A**) Proliferation rate changes in the lens after loss of *Mfn2* expression, as assessed by BrdU incorporation in the lens. Comparable numbers of lens cells are undergoing cell division in WT and *Mfn2 CKO* embryos, respectively, at E14.5, E16.5, E17.5, and E18.5. Magnified photo of E18.5 showed obvious difference between two groups. (**B**) Cell counts were documented according to a protocol modified from previous studies. (**C**) BrdU positive cells counts from E14.5 to E18.5. (**D**,**E**) The ratio of BrdU labeled cells was significantly reduced in *Mfn2 CKO* mice compared to controls after E17.5 (n = 5, *P < 0.05, **P < 0.001, ***P < 0.0001). Scale bars: 20 um (a–d), 40 um (e–h), 10 um (i and j).

To examine proliferation of lens epithelial cells, *in vivo* BrdU labeling was performed. The rate of BrdU incorporation from E14.5 to E16.5 was indistinguishable between the controls and *Mfn2 CKO* mice (Fig. 4A). After E17.5, the lens of *Mfn2 CKO* mice was smaller in size, and the labeled cells decreased in number and appeared in disarray within the lens, whereas most of the labeled cells were localized at the anterior lens epithelium in the controls (Fig. 4A). The absolute number of lens cells that incorporated BrdU was significantly (P < 0.05) reduced in *Mfn2 CKO* mice (Fig. 4C).

BrdU labeled cell ratio was significantly reduced in *Mfn2 CKO* mice compared to controls since E17.5 (Fig. 4C–E, n = 4, p < 0.05). Cell counts were conducted according to a protocol modified from previous studies (Fig. 4B)^21^. The BrdU labeling rate in lens epithelial cells was the lowest in the 0–15° sectors in both the *Mfn2 CKO* and wild-type mice, although there was no significant difference between the two groups (Fig. 4C–E). In this sector, cells begin to withdraw from the cell cycle for fiber cell terminal differentiation. The BrdU labeling rate was significantly decreased in central lens epithelial cells (60–90° sectors and 30–45° sectors) at E17.5 and E18.5 in the *Mfn2 CKO* mice compared to the wild-type (Fig. 4D,E).

### The absolute number of apoptotic lens cells in *Mfn2* knockout lens

**Figure 5:**
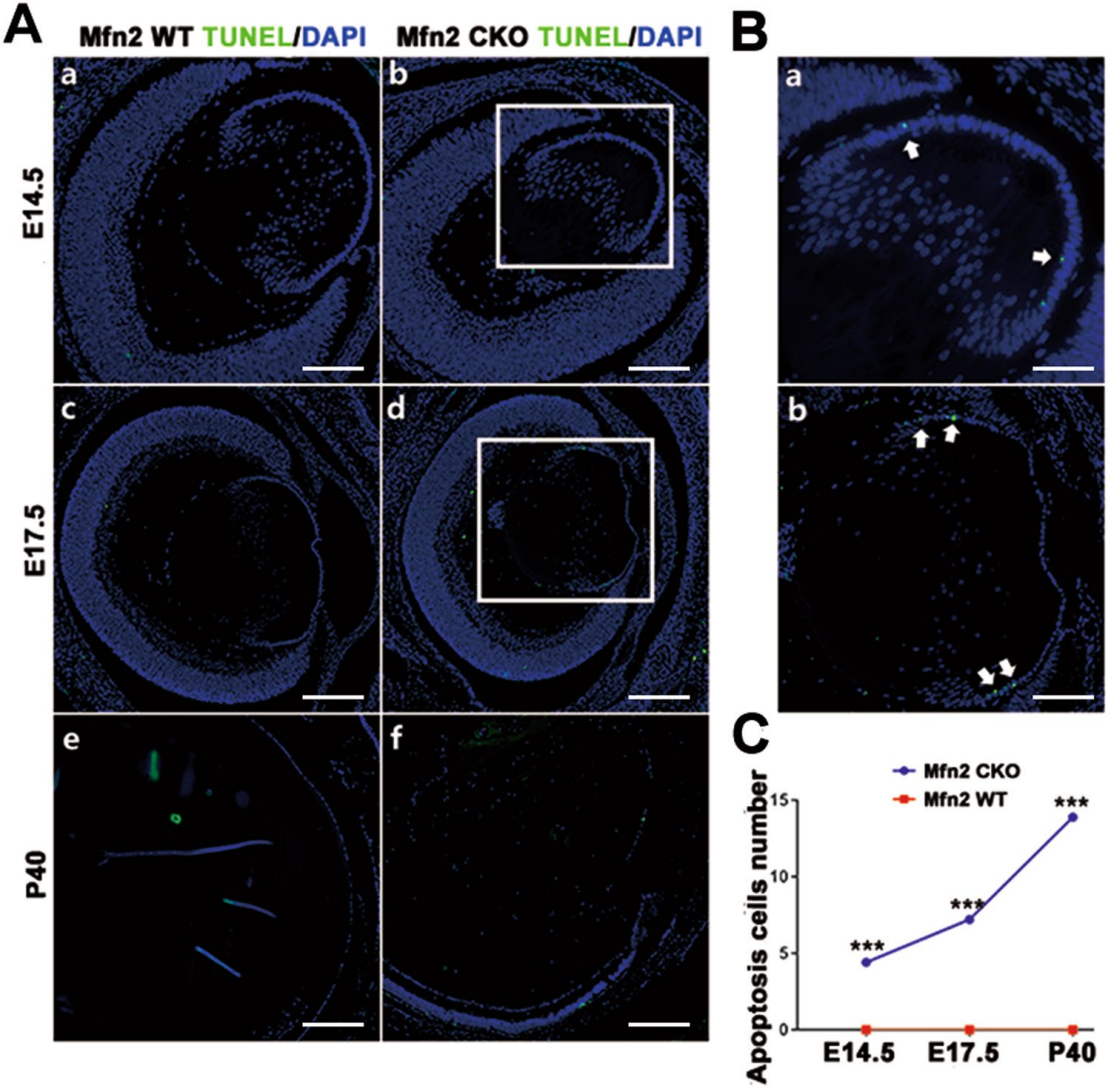
Apoptosis analysis in *Mfn2* knockout lens. (**A**) In *Mfn2 CKO* mice, apoptosis of LECs (arrows) was observed from E14.5 to be mainly concentrated near the equator. In P40, apoptotic cells were still detected in irregularly arranged lens cells of CKO mice (arrow); however, no apoptotic cells were found in the control group. (**B**) Magnified photo of (**A**,b) and (**A**,d). (**C**) The absolute number of lens apoptotic cells significantly (***P < 0.0001) increased in *Mfn2 CKO* mice. Scale bars: (**A**) 20 um (a,b) 40um (c,d), 200 um (e and f); (**B**) 10 um (a,b).

**Figure 6:**
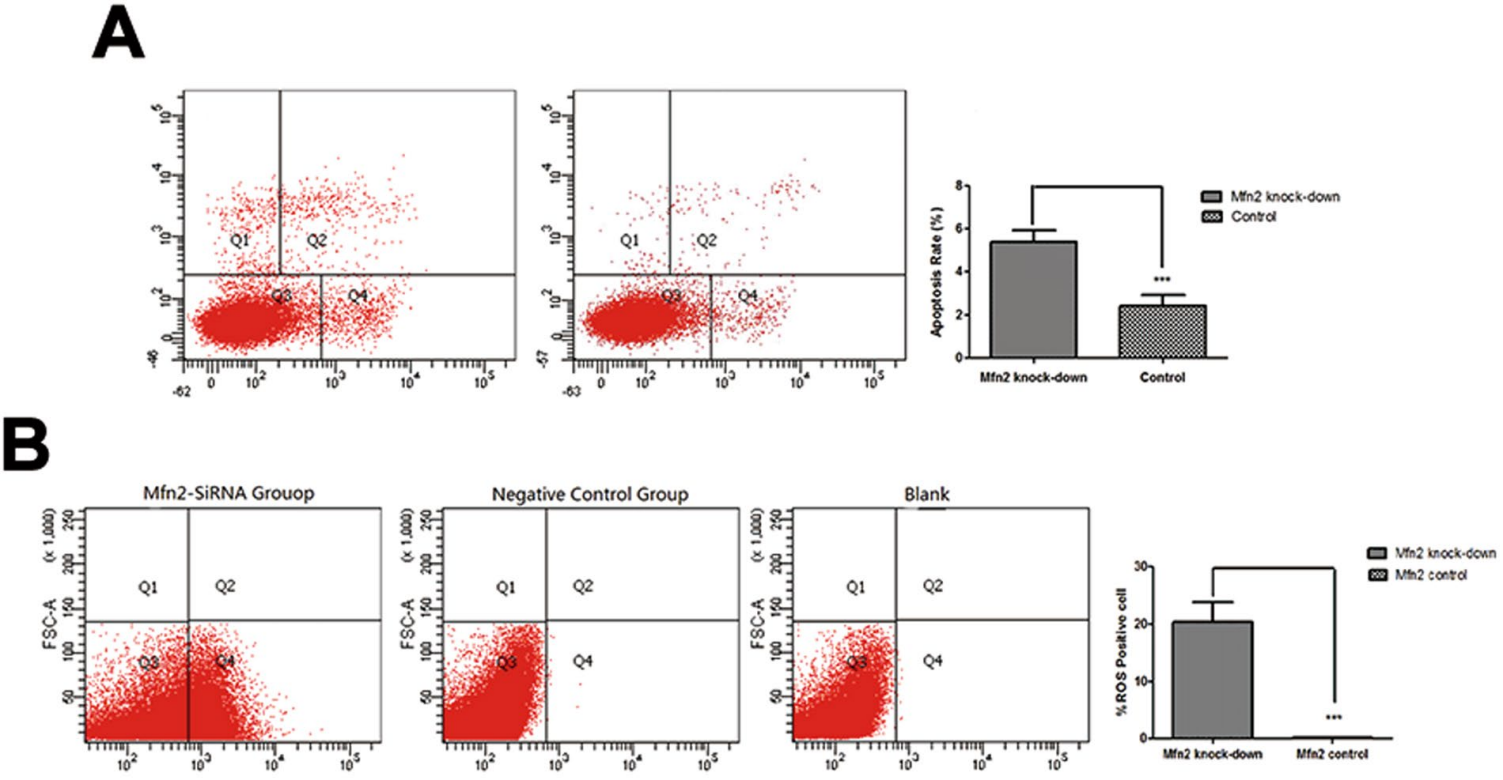
Apoptosis rate and ROS level in lens epithelium cell after Mfn2 gene was knockdown. (**A**–**C**) *Mfn2* gene was knocked down in cultured human SRA01/04 cells using si-RNA. For *in vitro* study, apoptosis rate of cultured human SRA01/04 cells increased by 2.32 times after *Mfn2* was knocked down by FACS. (**D**) Positive cells with increased ROS levels were significantly higher in the *Mfn2* knockdown group (P < 0.001).

To determine the reasons for abnormal lens development, the absolute number of apoptotic lens cells was counted. In *Mfn2 CKO* mice, apoptosis of LECs was observed since E14.5 and was mainly concentrated near the equator. Similar results were observed at E17.5 (Fig. 5A). In P40 apoptotic cells were still detected in irregularly arranged lens cells of CKO mice; however, no apoptotic cells were found in the control group (Fig. 5C). The absolute number of lens apoptosis cell was significantly (P < 0.01) increased in *Mfn2 CKO* mice (Fig. 5C). For *in vitro* study, after *Mfn2* knockdown in cultured human SRA01/04 cells using si-RNA, the apoptosis rate of cultured human SRA01/04 cells increased by 2.32 times (Fig. 6A).

### ROS in *Mfn2* knockdown lens epithelial cells

After *Mfn2* knockdown in cultured human SRA01/04 cells using si-RNA, ROS levels were measured by flow cytometry (Fig. 6B). Positive cells with increased ROS levels accounted for 0.88 ± 0.02% in the negative control group and 18.93 ± 1.33% in the *Mfn2* knockdown group, which represents a statistically significant difference (P < 0.001).

### Observation of LECs ultrastructure under transmission electron microscope

**Figure 7:**
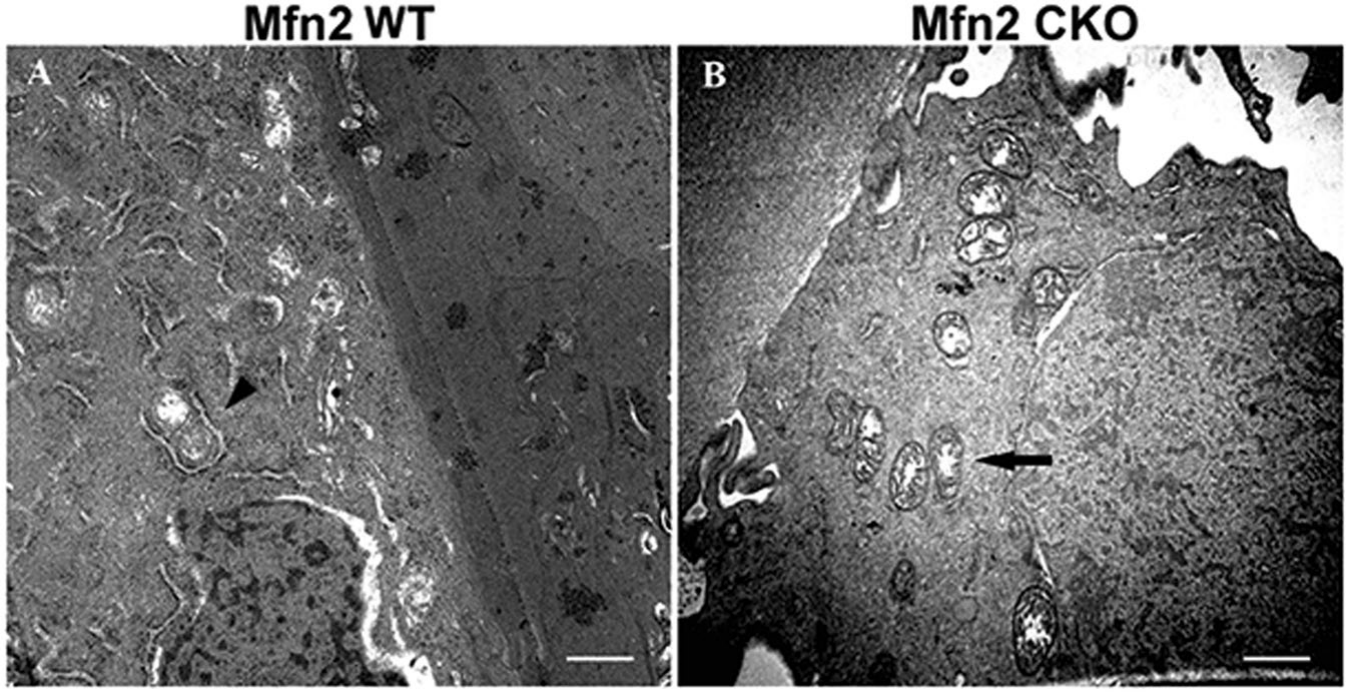
Lens epithelial cells ultrastructure under transmission electron microscope. (**A**) In wild type mice, the mitochondria in LECs were nearly round or ellipsoid, uniform in size, distributed around the nucleus, and encircled by abundant endoplasmic reticulum (arrow head). (**B**) In *Mfn2 CKO* mice, the mitochondria in LECs were not uniform in size, appeared oval. Portions of the mitochondria were found enlarged and filled with vacuoles. The endoplasmic reticulum adjacent to the mitochondria was loose and markedly reduced (arrow). Scale bars: 200 nm (**A**,**B**) (Magnified:20000X).

In the *Mfn2 WT* mice, the mitochondria in LECs were nearly round or ellipse, uniform in size, distributed around the nucleus, and encircled by abundant endoplasmic reticulum (Fig. 7A). There were no vacuoles between cells. In *Mfn2 CKO* mice, the mitochondria in LECs were not uniform in size, appeared oval shaped and distributed in areas surrounding the nucleus and in the cytoplasm. A portion of the mitochondria was enlarged and had vacuoles inside. The endoplasmic reticulum adjacent to the mitochondria was loose and markedly reduced, and there were abnormal vacuoles between cells (Fig. 7B).

## Discussion

Congenital cataract is a very common health problem in the cause of childhood blindness in the world. Human lens development requires a tightly regulated sequence of events and the interplay of many genes. Mutations in these genes negatively affect the structure and transparency of the lens, leading to cataracts. Although impaired mitochondria function could result lens opacity^11^, we have not seen any research in the literature which linking the congenital cataract to *Mfn2* gene malfunction. In this study, we used *Mfn2 CKO* mice to demonstrate that *Mfn2* is a significant contributor to lens transparency and eye development. Without the *Mfn2* gene at the head surface ectoderm, mice are born with congenital cataracts and microphthalmia. The effect of *Mfn2* conditional deletion on lens transparency was measured in *Mfn2 CKO* mice by comparison with age-matched WT mice. As shown in Fig. 1, the lenses were clear in WT mice at 3 months of age, but the lenses of CKO animals exhibited severe cataract at 3 months, indicating rapid loss of transparency after birth. We used a clinical slit lamp and a quantitative LOCS III cataract grading system for this study. Our model showed a very early onset of lens opacity at birth. The presence of cataract in the *Mfn2 CKO* mice was near 84% at the time of eyelid opening. The *Mfn2 CKO* mice also displayed a rapid and progressive increase in lens opacity, with a 3.75 score as early as 1 months of age, and a 9.83 higher score by the age of 3 months. Examination of histological sections revealed that lens of *Mfn2 CKO* mice embryo appeared to be slightly smaller, but the complete structure was fully developed. The eyes of *Mfn2 CKO* mice showed a smaller lens with slight opacity, and cortex vacuole in the central lens after birth. lens opacity and the size of the cortex vacuole become more severe with age.

Previous studies have established that *Mfn2* can mediate the fusion of mitochondria and thereby contribute to the dynamic balance between fusion and fission that determines mitochondria morphology. *Mfn2* expression was detected in many tissues and cell lines, with the highest expression found in the heart and skeletal muscle, lower levels in the brain, kidney, and liver^22^. However, there are no literature reports of detailed *Mfn2* expression in the eye. Our study shows that during lens development, Mfn2 transcripts were first detected at embryonic day 10. After that, *Mfn2* transcripts were present in low levels in lens epithelial cells and at moderate to high levels in primary lens fiber cells, and such expression was weak in the proliferating lens epithelium. This dynamic expression pattern suggests that *Mfn2* expression is strictly regulated during eye development, especially in differentiated lenses.

A previous study found that knockdown of *Mfn2* expression in muscle cells lead to fragmentation of the mitochondrial network into independent clusters^22,23^. *Mfn2* is found to be abundant at the endoplasmic reticulum (ER)-mitochondria interface. Ablation or silencing of *Mfn2* in mouse embryonic fibroblasts and HeLa cells disrupted ER morphology and loosened ER-mitochondria interactions^24,25^. To elucidate the changes of ER and mitochondria in LECs when *Mfn2* was conditional knockout, we investigated the ultrastructure of LECs of *Mfn2 CKO* mice. We found that the mitochondria in LECs were not uniform in size and part of the mitochondria was swollen, enlarged and contained vacuoles inside. The ER adjacent to the mitochondria was markedly reduced. These ultrastructural changes indicate that *Mfn2* conditional knockout affects the morphological stability of mitochondria and may lead to lysis of ER surrounding the mitochondria. This suggests that ER-mitochondria contacts may also be influenced, although this will require further study to confirm.

A previous study also found that *Mfn2* is a primary determinant of vascular smooth muscle cell apoptosis^24^. Kasahara found that ablation of the mitochondrial fusion proteins Mfn1 and Mfn2 in the embryonic mouse heart, or gene trapping of *Mfn2* or optic atrophy-1 in mouse embryonic stem cells, arrested mouse heart development and impaired differentiation of embryonic stem cells into cardiomyocytes^26^. In this study, we found that conditional knockout of *Mfn2* gene expression affected lens cell proliferation and apoptosis during lens development. At late stage of lens development, lens epithelial cell proliferation decreases, especially at the anterior part of the lens. This is one of the causes of lens maldevelopment and small eye. However, we observed an evident increase in LECs apoptosis during lens development, while there was no such finding in normal control mice. Abnormal apoptosis of LECs was found since E14.5 and was mainly concentrated near the equator. *In vitro* study found that after *Mfn2* knockdown, the apoptosis rate of human LECs in the *in vitro* culture increased by more than 2 folds. As some study reported, the main contributor of cataract development is abnormal apoptosis of LECs, which causes lens opacity^27,28^. *Mfn2* conditional knockout also leads to abnormal apoptosis of LECs and thus induces cataracts. Apoptosis may be affected by many factors, including oxidative stress. Oxidative stress plays a significant role in cataractogenesis^11^. The lens is highly susceptible to ROS, and the mitochondria have been confirmed as a major source of ROS generation^29^. Several *in vitro* studies have demonstrated that human lens cells are highly susceptible to oxidative insults in which antioxidant activity is generally inversely proportional to cataract severity^30,31^. The *in vitro* experiment in the present study proved that after *Mfn2* knockdown in human LECs, ROS significantly increased to as high as 18.9%, while the percentage in the control group was nearly 0. At day >14, the eyelids of *Mfn2 CKO* mice were opened and the lenses were more exposed to exogenous stimulation, such as light and ultraviolet rays. Without *Mfn2* protein in lens epithelial cell, increased ROS may accelerate apoptosis of LECs and impair the function of mitochondria, leading to notable vacuolization of the lens.

According to the literature, mitochondrial morphology and fusion and fission dynamics controlled by mitofusins directly influence mitochondrial metabolism, apoptotic and necrotic cell death^1,3,6,19^. But all these are assumptions and need further investigation. One research mentioned that eye abnormalities can occur in hemizygous Le-Cre^Tg/−^ mice on CBA/Ca genetic backgrounds, in the absence of a floxed allele, such as small lens, malformed lens, vacuolated lens, persistent lens stalks. Moreover, the eye abnormalities diminished after backcrossing Le-Cre^Tg/-^ mice to the original FVB/N strain for two generations^32^. In the present study, we crossed the Le-Cre mice with C57/B6 mice and didn’t find eye abnormality for ten generations. Furthermore, *Mfn2 CKO* mouse corneas became thickened, and eyeballs appeared slightly smaller before delivery. The phenotype was differed from the published literature.

In summary, this morphologic study provides the first direct genetic evidence of the role of *Mfn2* in congenital cataracts and microphthalmia, delineating the distinct function of Mfn2 in regulating lens growth and development. The observed changes in proliferation and apoptosis further support the importance of *Mfn2* in eye development. Further investigation of *Mfn2* and mitochondria metabolism, mtDNA stability and various signaling pathway molecules in directing growth and morphogenetic events in the developing eye should clarify the mechanism of *Mfn2* gene function in eye development. We suggest that genetic testing of *Mfn2* mutations should be included for patients with a clinical diagnosis of congenital cataracts in the future to corroborate our findings in this animal study.

## Methods

### Experimental mouse breeding and genotyping

All animal experiments and all of relevant details, including experiments protocols were performed in accordance with approved guidelines and regulations established by the University of Southern California, Zhejiang University and China Medical University. The mice used in this study were housed in a controlled, specific pathogen-free (SPF) environment and cared for according to the approved protocol. Experimental mice used in this study carry floxed *Mfn2* alleles^13^ and Le-Cre mice^32^. Timed matings were performed between female *Mfn2fl/fl* mice and male *Mfn2*+/*fl/Le-Cre*+ mice to generate *Mfn2 CKO* mice (*Mfn2fl/fl/Le-Cre*+) and control mice (*Mfn2fl*/+ or *Mfn2fl/fl*). The floxed Mfn2 (*Mfn2fl/fl*) mice were provided by Dr. Yi Hsin-Liu (Doheny Eye Institute, USA) with permission from Dr. DC Chan (California Institute of Technology, USA). *Le-Cre* heterozygous knockin (Le-Cre+/−) mice were obtained from David Beebe (Washington University, St. Louis, USA) with permission from Peter Gruss (Max-Planck Institute for Biophysical Chemistry, Germany) and Ruth Ashery-Padan (Tel Aviv University, Israel). All mice were bred in a C57BL/6 strain background. Genotypes were determined using the tail tissue genomic DNA PCR method^33^. PCR primers for *Mfn2* genotyping were 5′-GAA GTA GGC AGT CTC CAT CG-3′ and 5′-AAC ATC GCT CAG CCT GAA CC-3′. PCR primers used to detect Cre knock-ins were 5′-CTC TGG TGT AGC TGA TGA TC-3′ and 5′-TAA TCG CCA TCT TCC AGC AG-3′.

### Western Blot

Protein of Lens capsule and lens cells of *Mfn2 CKO* mice and control at P60 were collected for confirming whether Mfn2 was conditional knockout or not. Protein was extracted by Minute TM Total Protein Extraction Kit for Animal Cultured Cells and Tissues (Invent Biotechnologies, Inc., USA). 200 μl native lysis buffer was added to the filter and the grinding continued for 30–60 times. After tissue was grinded, the tube was incubated on ice for 5 min and centrifuged at top speed for 1–2 min and protein quantification were performed. Protein electrophoresis was performed following standard protocol.

### Determining the rate of cataract formation and opacity scoring

The present study used an *Mfn2 CKO* mouse (*Mfn2*^*fl/fl*^*/Le-Cre*^+^) model. The lenses in *Mfn2 CKO* mice and age-matched wild-type (WT) mice from the same background were examined and imaged using a slit lamp microscope (66 Instrument Co, Suzhou, PRC) equipped with a Sony digital camera after the pupils were dilated with tropicamide phenylephrine eye drops (Santen Pharmaceutical Co., Ltd., Japan) without anesthetization. Mice at ages 1, 2, and 3 months old were used in this study. We observed 12 mice (24 eyes) in each group for assessing rate of cataract formation by using slit lamp after mydriasis. Specifically, the number of eyes obtained from *Mfn2 CKO* mice at various ages were 12, 10 and 10 at 1, 2 and 3 months, respectively. The same number of eyes were obtained from wild-type mice at the same time points. The LOCS III system^34^ was used for cataract classification and grading. The cataract score reported in this study was calculated based on the sum nuclear, cortex and posterior subcapsular opacity scores divided by the total number of eyes examined.

### BrdU labeling, isolation and preparation of mouse embryos and eyes

Pregnant female mice were sacrificed at various time points after conception. One hour before sacrificing, the mice were injected intraperitoneally with 100 µg BrdU (Sigma, St. Louis, USA) per gram of body weight. The animals were then sacrificed, and embryos were dissected and isolated in ice-cold PBS. A piece of tail tissue was taken from each embryo for DNA extraction and genotyping. Embryos or eyeballs were then fixed in 4% paraformaldehyde (PFA) in PBS or fresh Davidson’s fixative (33% ethanol, 22% formaldehyde, 11% glacial acetic acid) overnight at 4 °C.

### Histology

For histological analysis, embryos or eyeballs were further dehydrated through graded alcohol, cleared in xylene, and embedded in paraffin. Sections were cut to 4 µm for immunohistochemistry and hematoxylin and eosin staining as reported previously.

### *In situ* hybridization

For *in situ* hybridization analysis, the fixed embryos were rinsed in PBS for 10 minutes then cryoprotected in 30% sucrose overnight. Embryos were oriented in OCT compound (Sakura Finetek, Torrance, USA) and rapidly frozen. Sections were cut to 12 µm and mounted on Superfrost Plus glass slides (Thermofisher, CA, USA) for future experiments. *In situ* hybridization was performed as previously described^35^. *Mfn2* RNA probes were assembled by PCR and labeled with digoxigenin-UTP according to the manufacturer’s recommendations (Roche Applied Science, Indianapolis, USA). The reactions were revealed by immunocytochemistry using an anti-digoxigenin-AP Fab fragment antibody (Roche Applied Science, Indianapolis, USA). Sections were photographed using an Olympus microscope (BX51, Olympus, Japan) with a SPOT camera.

### Immunohistochemistry for lens cell proliferation assay

Fixed sections were rehydrated. Endogenous peroxidases were blocked with 3% H_2_O_2_. Epitope retrieval was performed in a 0.1 M sodium citrate buffer (pH 6.8) at 100 °C for 10 minutes before adding blocking reagents. After the addition of primary antibodies, sections were incubated in a humidified chamber at 4 °C overnight. Mouse anti-BrdU (Sigma, St. Louis, USA) primary antibodies were used. Fluorophore-labeled anti-mouse IgG antibody (Invitrogen, Carlsbad, USA) was used for signal acquisition.

### Transmission electron microscope

At day 15 after birth, the lenses of mice of the *Mfn2 CKO* and control groups were collected and fixed for use. After conventional fixation and preparation of samples for transmission electron microscopy, ultrastructural changes in equatorial LECs from two groups were observed.

### Cell culture

SRA01/04 human lens epithelium cells (LECs) line^36^ were gifted from Dr. Yi-sin Liu (Doheny Eye Institute, USA). SRA01/04 cells were cultured in Dulbecco’s Modified Eagle’s Medium (DMEM) (Invitrogen, Carlsbad, USA) supplemented with 10% fetal bovine serum (FBS) (Hyclone, Logan, USA), 100 U/ml penicillin, and 100 U/ml streptomycin. Cells were incubated in a humidified 37 °C incubator containing 5% CO_2_. SRA01/04 cells were transfected using Lipofectamine 2000 (Invitrogen, Carlsbad, USA) according to the manufacturer’s protocol. Small interfering RNA (siRNA) for *Mfn2* and a negative control were designed and synthesized by GenePharma (Shanghai, PRC) and transfected into SRA01/04 cells. *Mfn2* siRNA sequences used were 5′-GGAAGAGCACCGUGAUCAATT-3′ (sense) and 5′-UUGAUCACGGUGCUCUUCCTT-3′ (antisense). After 48 hours, *Mfn2* expression was tested by RT-qPCR and Western blot. Cell apoptosis rate and intracellular ROS were measured by flow cytometry.

### Lens cell apoptosis assay *in vivo* and *in vitro*

Apoptotic cells in mouse embryos were detected *in vivo* using the fluorescein *in situ* Cell Death Detection Kit (Roche Applied Science, Indianapolis, USA). Briefly, 4% PFA-fixed tissue sections were boiled for 10 minutes. Fragmented DNA was labeled with fluorescein-dUTP using terminal transferase. Fluorescence and bright-field images of the sections were taken using an Olympus microscope (BX51, Olympus) with a SPOT camera.

*In vitro* apoptosis was determined based on the counts of apoptotic cells in the cultured SRA01/04 cell line after *Mfn2* knockdown by siRNA. Apoptosis was determined using a dead cell apoptosis kit with annexin V and PI for flow cytometry (Invitrogen, Carlsbad, USA). After incubation for 15 min at room temperature in the dark, specimens were assessed by flow cytometry (BD Biosciences, USA). Assays were performed for three independent experiments.

### ROS detection

At 48 h after transfection, SRA01/04 cells (1–5 × 10^5^/well) were collected, and intracellular ROS levels in control and *Mfn2* knockdown cells were evaluated by loading with 10 ug/ml of 2′7′-dichlorofluorescin (DCFH-DA, Sigma, St. Louis, USA) and incubation at 37 °C for 20 min. DCF fluorescence was measured using flow cytometry.

### Statistical Analysis

Data were recorded as mean ± standard deviation (SD), and analyzed using SPSS for Windows, version 16.0 (SPSS Inc. IL, USA). Significant difference was evaluated by analysis of unpaired Student’s -test (two-tailed). Statistical significance was defined as *P* < 0.05.

## Electronic supplementary material

supplementary material

## References

[1] Kasahara A, Scorrano L (2014). Mitochondria: from cell death executioners to regulators of cell differentiation. Trends Cell Biol..

[2] Kasahara A, Cipolat S, Chen Y, Dorn GW, Scorrano L (2013). Mitochondrial fusion directs cardiomyocyte differentiation via calcineurin and Notch signaling. Science..

[3] Fischer F, Hamann A, Osiewacz HD (2012). Mitochondrial quality control: an integrated network of pathways. Trends Biochem Sci..

[4] Gomes LC, DiBenedetto G, Scorrano L (2011). During autophagy mitochondria elongate, are spared from degradation and sustain cell viability. Nat Cell Biol..

[5] Hamanaka RB (2013). Mitochondrial reactive oxygen species promote epidermal differentiation and hair follicle development. Sci Signal..

[6] Chen Y, Liu Y, Dorn GW (2011). Mitochondrial fusion is essential for organelle function and cardiac homeostasis. Circ Res..

[7] Alavi MV (2007). A splice site mutation in the murine Opa1 gene features pathology of autosomal dominant optic atrophy. Brain..

[8] Davies VJ (2007). Opa1 deficiency in a mouse model of autosomal dominant optic atrophy impairs mitochondrial morphology, optic nerve structure and visual function. Hum. Mol. Genet..

[9] Reeve AK, Krishnan KJ, Turnbull D, Mitochondrial DNA (2008). mutations in disease. aging, and neurodegeneration. Ann NY Acad Sci..

[10] Jarrett SG, Lin H, Godley BF, Boulton ME (2008). Mitochondrial DNA damage and its potential role in retinal degeneration. Prog Retin Eye Res..

[11] Jarrett SG, Lewin AS, Boulton ME (2010). The importance of mitochondria in age-related and inherited eye disorders. Ophthalmic Res..

[12] Santel A, Fuller MT (2001). Control of mitochondrial morphology by a human mitofusin. J. Cell Sci..

[13] Chen H (2003). Mitofusins Mfn1 and Mfn2 coordinately regulate mitochondrial fusion and are essential for embryonic development. J Cell Biol..

[14] Filadi R (2015). Mitofusin 2 ablation increases endoplasmic reticulum-mitochondria coupling. Proc Natl Acad Sci USA.

[15] Gall JM (2015). Conditional knockout of proximal tubule mitofusin 2 accelerates recovery and improves survival after renal ischemia. J Am Soc Nephrol..

[16] Kawalec M (2015). Mitofusin 2 Deficiency Affects Energy Metabolism and Mitochondrial Biogenesis in MEF Cells. PLoS One..

[17] Sawyer SL (2015). Homozygous mutations in MFN2 cause multiple symmetric lipomatosis associated with neuropathy. Hum Mol Genet..

[18] Jiang B (2017). Identification of a novel missense mutation of MIP in a Chinese family with congenital cataracts by target region capture sequencing. Sci Rep..

[19] Chen H, McCaffery JM, Chan DC (2007). Mitochondrial fusion protects against neurodegeneration in the cerebellum. Cell..

[20] Chen H (2010). Mitochondrial fusion is required for mtDNA stability in skeletal muscle and tolerance of mtDNA mutations. Cell..

[21] Rajagopal R (2008). Functions of the type 1 BMP receptor Acvr1 (Alk2) in lens development: cell proliferation, terminal differentiation, and survival. Invest Ophthalmol Vis Sci..

[22] Bach D (2003). Mitofusin-2 determines mitochondrial network architecture and mitochondrial metabolism. A novel regulatory mechanism altered in obesity. J Biol Chem..

[23] Santel MTF (2001). Control of mitochondrial morphology by a human mitofusin A. Journal of Cell Science..

[24] Guo X (2007). Mitofusin 2 triggers vascular smooth muscle cell apoptosis via mitochondrial death pathway. Circ Res..

[25] De B, Scorrano OM (2008). L. Mitofusin 2 tethers endoplasmic reticulum to mitochondria. Nature..

[26] Yan Q, Liu JP, Li DW (2006). Apoptosis in lens development and pathology. Differentiation..

[27] Zhang L (2010). Apoptosis: its functions and control in the ocular lens. Curr Mol Med..

[28] Huang L, Tang D, Yappert MC, Borchman D (2006). Oxidation-induced changes in human lens epithelial cells 2. Mitochondria and the generation of reactive oxygen species. Free Radic Biol Med..

[29] Pescosolido N, Barbato A, Giannotti R, Komaiha C, Lenarduzzi F (2016). Age-related changes in the kinetics of human lenses: prevention of the cataract. Int J Ophthalmol..

[30] Varma SD, Kovtun S, Hegde KR (2011). Role of ultraviolet irradiation and oxidative stress in cataract formation-medical prevention by nutritional antioxidants and metabolic agonists. Eye Contact Lens..

[31] Dorà NJ, Collinson JM, Hill RE, West JD (2014). Hemizygous Le-Cre transgenic mice have severe eye abnormalities on some genetic backgrounds in the absence of LoxP sites. PLoS One..

[32] Ashery-Padan R, Marquardt T, Zhou X, Gruss P (2000). Pax6 activity in the lens primordium is required for lens formation and for correct placement of a single retina in the eye. Genes Dev..

[33] Truett GE (2000). Preparation of PCR-quality mouse genomic DNA with hot sodium hydroxide and tris (HotSHOT). Biotechniques..

[34] Chylack LT (1993). The Lens Opacities Classification System III. The Longitudinal Study of Cataract Study Group. Arch Ophthalmol..

[35] Li M (2003). Expression of murine ELL-associated factor 2 (Eaf2) is developmentally regulated. Dev Dyn..

[36] N. Ibaraki SC (1998). Human lens epithelial cell line. Exp Eye Res..

