# Supplementary material for: Embryonic Surface Ectoderm-specific Mitofusin 2 Conditional Knockout Induces Congenital Cataracts in Mice
Source: Sci Rep. 2018 Jan 24;8:1522. doi: 10.1038/s41598-018-19849-2 (PMC5784114; doi:10.1038/s41598-018-19849-2)
Supplement: Supplementary file 1 — supplementary material [file 41598_2018_19849_MOESM1_ESM.pdf]

Supplementary material

Embryonic Surface Ectoderm-specific Mitofusin 2 Conditional Knockout Induces Congenital Cataracts in Mice

Jiangyue Zhao[1,2], Xinwei Wu[2], Danhong Wu[3], Yinhui Yu[1], Yibo Yu[1], Yao Wang[1], Qiuli Fu[1], Jinsong Zhang[2], Ke Yao[1*].

[1] The Department of ophthalmology, Eye center of the 2nd Affiliated Hospital, Medical College of Zhejiang University, Hangzhou 310009, China

[2] The Department of ophthalmology of the 4thAffiliated Hospital, China Medical University, Shenyang 110005, China

[3] Department of Neurology, Shanghai fifth People's Hospital, Fudan University, Shanghai 200240, China

*Correspondence: Ke Yao, Department of ophthalmology, Eye center of the 2nd Affiliated Hospital, Medical College of Zhejiang University, Hangzhou 310009, China; xlren@zju.edu.cn

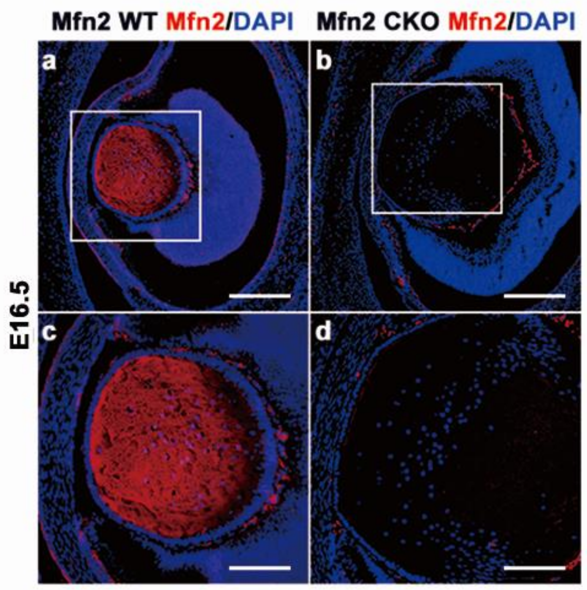

Supplementary figure legend

Figure 1. Mfn2 was conditional knockout in lens. At E16.5d, Mfn2 expression was found in lens fiber cells of Mfn2 WT mice but diminished in Mfn2 CKO mice.

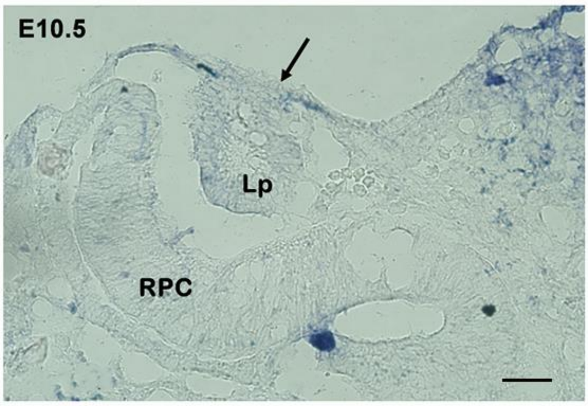

Figure 2. Normal Mfn2 transcript spatiotemporal location during lens development. Mfn2 transcript were present in low level at lens placode and retina progenitor cell (RPC) at E10.5. Scale bar:100

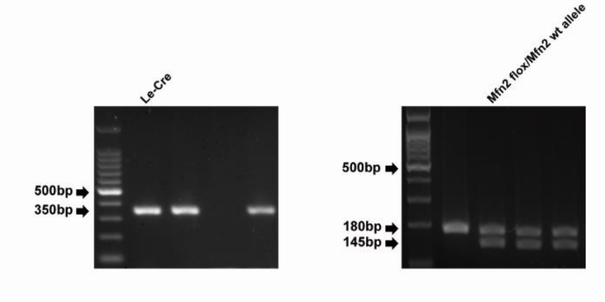

Figure 3. Full-length gel of mice tail PCR genotyping. Detection of Le-Cre and Mfn2 allele by genotyping PCR. The fragment of 350 bp indicats Le-Cre, the fragment of 180 bp indicates Mfn2 floxed allele and the 145bp fragment indicates wild-type Mfn2 gene.
